# Giant electrocaloric response in smectic liquid crystals with direct smectic-isotropic transition

**DOI:** 10.1038/s41598-019-38604-9

**Published:** 2019-02-11

**Authors:** Eva Klemenčič, Maja Trček, Zdravko Kutnjak, Samo Kralj

**Affiliations:** 1 Faculty of Natural Sciences and Mathematics, Koroška cesta 160, 2000 Maribor, Slovenia; 2 Jozef Stefan Institute, Jamova 39, 1001 Ljubljana, Slovenia; 3 The Jozef Stefan International Postgraduate School, Jamova 39, 1001 Ljubljana, Slovenia

## Abstract

Electrocaloric materials have become a viable technology for solid state heat management applications. We demonstrate both theoretically and experimentally that liquid crystals (LCs) can be exploited as efficient electrocaloric materials. Numerical and experimental investigations determine the conditions under which the strongest electrocaloric effect (ECE) responses are expected in LCs. Specifically, we show that a large ECE can be expected at the isotropic-nematic and in particular at the isotropic-smectic A phase transition. In our theoretical study, LC ordering is modelled using a Landau – de Gennes - Ginzburg mesoscopic approach. The simulation results are in qualitative agreement with our high precision electrocaloric measurements conducted on 8CB and 12CB liquid crystals. In the latter, we obtained Δ*T*_EC_ ~ 6.5 K, corresponding to the largest response measured so far in LCs. The fluid property of LC electrocaloric heat cooling elements could lead to the development of devices with a higher coefficient of performance and thus better cooling power yield per mass of the ECE-based device.

## Introduction

The electrocaloric effect^1,2^ is related to a reversible change in the temperature Δ*T*_EC_ of a material upon switching an electric field on or off under adiabatic conditions. The switching process is fast enough so that within it, negligible heat is exchanged between the electrocaloric material and the surrounding thermal bath. Studies of various ferroelectric materials have revealed anomalously giant ECE responses^3–14^. These indicate that ECE has potential for numerous applications, particularly in heating and cooling or heat waste recovery devices^6,15–18^. These are expected to provide an efficient alternative to applications based on mechanical vapour compression cycle, thermoelectric, or other caloric effects. ECE based devices have potential for miniaturization that can be exploited to develop efficient cooling mechanisms in computer devices. Furthermore, in contrast to classical cooling technologies that rely on environmentally dangerous gases, ECE cooling devices will be environmentally friendlier.

In order to develop commercially competitive ECE-based applications one needs to find adequate electrocaloric (EC) materials that experience sufficient electrocaloric temperature responses, of the order of ${\rm{\Delta }}{T}_{{\rm{EC}}}\sim 10\,{\rm{K}}$, for moderate changes in an external electric field *E*. Note that this difference could be further enhanced by an order of magnitude by using active regenerator approaches^19^. For this purpose, one needs EC material exhibiting a relatively large change in entropy on varying *E*. In particular, this could be realized near a symmetry breaking order-disorder phase transition in which an order parameter field spontaneously appears in the lower symmetry phase. Recent experiments suggest that the EC response near a first order phase transition is proportional with the latent heat *L* released or adsorbed. Therefore, materials exhibiting relatively large values of *L* are desirable. Furthermore, the phase transition temperature should increase with increasing *E*. In such a way one could trigger ordering on increasing *E* starting from a disordered state. Recent experimental studies^20–25^ reveal that liquid crystals (LCs) may be appropriate for this purpose.

LC phases and structures are formed by relatively weakly interacting anisotropic molecules^26^. In general, their phases exhibit long range orientational, and in some cases, (quasi) long range translational ordering. They display a rich diversity of phases and structures, several of which are stable at room temperatures, in which entropic interactions play a significant role. LCs combine an unique combination of order, liquid character and softness. In addition, several LC phases and structures possess a relatively strong dielectric anisotropy or permanent electrical dipoles. Softness refers to the ability of LCs to yield a strong macroscopic response in ordering, even to weak external stimuli. This property is inherent in LCs because most of their phases or structures are reached via a continuous symmetry breaking phase transition. Note that the order parameter field of any continuous breaking phase transition generally consists of two qualitatively different components: the *amplitude* field and the *symmetry breaking* (also commonly referred to as the *gauge*) field. The *amplitude* field determines the degree of established ordering and equals zero in the higher symmetry phase. The *gauge* field reveals the symmetry breaking choice in the lower symmetry phase. The *gauge* field exhibits symmetry restoring Goldstone excitations which endow LCs with softness^26^.

In our study, we consider thermotropic n-alcyl cyanobiphenyl (nCB) LCs consisting of rod-like molecules exhibiting positive dielectric anisotropy Δ*ε*. Of interest is nematic (N) and smectic A (SmA) LC ordering. The uniaxial nematic phase is the simplest LC phase, possessing only orientational ordering. A local orientation is commonly determined at the mesoscopic scale by the nematic director field $\mathop{n}\limits^{\rightharpoonup }$, corresponding to the *gauge* field component of the nematic order parameter. It exhibits head-to-tail invariance (*i*.*e*., states ±$\mathop{n}\limits^{\rightharpoonup }$ are physically equivalent). In bulk equilibrium nematic long range ordering is spatially homogeneous, where $\mathop{n}\limits^{\rightharpoonup }$ is aligned along a single symmetry breaking direction. In the SmA phase, additional translational ordering appears. In bulk equilibrium, this consists of parallel stacks of equidistant smectic layers, where $\mathop{n}\limits^{\rightharpoonup }\,$ is aligned along the layer surface normal. Due to effectively two-dimensional layer ordering, the SmA phase exhibits quasi long-range ordering. LC phases are reached by lowering temperature *T* from the isotropic (I), ordinary liquid phase. The sequence of phases depends on the length of aliphatic chains of anisotropic nCB molecules.

The dominant interaction between nCB molecules and an external electric field *E* exhibits a quadrupolar character, tending to align $\mathop{n}\limits^{\rightharpoonup }$ parallel to $\mathop{E}\limits^{\rightharpoonup }$. This leads to a reduction in dipolar entropy contribution. The largest *E* - driven changes in the latter component are expected close to I-N and I-SmA order-disorder phase transitions, in which orientational order appears. Indeed, preliminary experimental results performed by Lelidis and Durand^27^ and Zhang’s group^28^ indicate that ECE at the I-N transition could be substantial.

In this paper we consider LCs as dielectric materials. Our aim is to determine conditions (temperature regime and material properties) for which one could obtain maximal ECE-driven temperature response Δ*T*_EC_ if an external field *E* is switched on or off. For this purpose, we need to maximize *E* driven change in LC orientational entropy contribution (i.e. in LC orientational ordering). We use a minimal mesoscopic Landau-de Gennes-Ginzburg type modelling to determine key parameters controlling ECE at the I-N and I-SmA phase transition. We test the validity of our modelling by measuring Δ*T*_EC_ in 8CB and 12CB LCs, which exhibit the I-N and direct I-SmA phase transition, respectively.

## Results

We identify key parameters affecting electrocaloric driven temperature changes Δ*T*_EC_ in nCB LCs. For this purpose, one needs to consider external electric field *E* driven entropy changes. In an adiabatic process, the total entropy is conserved, i.e. ${{\rm{\Delta }}{\rm{\Omega }}}_{tot}$ = ${{\rm{\Delta }}{\rm{\Omega }}}_{{\rm{LC}}}+{{\rm{\Delta }}{\rm{\Omega }}}_{l}$ = 0. Here, ${{\rm{\Delta }}{\rm{\Omega }}}_{tot}$ stands for the total change in entropy, ${{\rm{\Delta }}{\rm{\Omega }}}_{{\rm{LC}}}$ = ${{\rm{\Omega }}}_{{\rm{LC}}}[{E}_{2},{T}_{2}]-{{\rm{\Omega }}}_{{\rm{LC}}}[{E}_{1},{T}_{1}]$ determines the change in LC orientational degrees of freedom, and ${{\rm{\Delta }}{\rm{\Omega }}}_{l}$ = ${{\rm{\Omega }}}_{l}[{T}_{2}]-{{\rm{\Omega }}}_{l}[{T}_{1}]$ labels the entropy change in the so-called lattice contribution. The latter contribution refers to lattice vibrations within the system. The subscripts “1” and “2” refer to the initial state {*E*_1_, *T*_1_} and the final state {*E*_2_, *T*_2_} of the adiabatic process, respectively.

We calculate Ω_LC_ within the volume *V* for given conditions from the expression

1 $${{\rm{\Omega }}}_{{\rm{LC}}}[E,T]=-\,{[\partial {F}_{{\rm{LC}}}/\partial T]}_{E},$$

where *F*_LC_ describes the free energy contribution of LC orientational degrees of freedom. On the other hand, we express the lattice contribution from the relation

2 $${{\rm{\Delta }}{\rm{\Omega }}}_{l}={\int }_{{T}_{1}}^{{T}_{2}}\,\frac{{C}_{l}[T]}{T}dT\sim {C}_{l}\,{\int }_{{T}_{1}}^{{T}_{2}}\,\frac{dT}{T}\sim {C}_{l}\frac{{\rm{\Delta }}{T}_{{\rm{EC}}}}{{T}_{1}}.$$

Here *C*_*l*_ stands for the heat capacity contribution per volume of the remaining degrees of freedom in which we neglect temperature variations within the temperature interval $[{T}_{1},{T}_{2}={T}_{1}+{\rm{\Delta }}{T}_{{\rm{EC}}}]$.

### Modelling of LC phase behaviour

In expressing *F*_LC_, we use the Landau-de Gennes-Ginzburg approach in terms of the nematic tensor order parameter **Q** and the smectic complex order parameter $\psi =\eta {e}^{i\varphi }$. In the case of nematic uniaxial ordering, **Q** can be described with the nematic director field $\mathop{n}\limits^{\rightharpoonup }$ and the uniaxial order parameter *S*. The unit vector $\mathop{n}\limits^{\rightharpoonup }$ points along a local uniaxial direction. The uniaxial nematic order parameter *S* describes the amplitude of orientational ordering. In a perfectly aligned nematic phase and isotropic phase, it holds *S* = 1 and *S* = 0, respectively. The translational order parameter *η* quantifies the degree of translational ordering and the phase $\varphi$ locates the smectic layers.

In our modelling we assume that the *amplitude* fields (*S* and *η*) of the order parameters are spatially homogeneous and that the respective *gauge* fields ($\mathop{n}\limits^{\rightharpoonup }$ and $\varphi$) are spatially undistorted. Therefore, $\mathop{n}\limits^{\rightharpoonup }$ is spatially homogeneously aligned along a single symmetry breaking direction (say along the z-axis of the Cartesian coordinate system (*x*,*y*,*z*)) and smectic layers of thickness *d* are equidistant, determined by $\varphi =d=2\pi z/d$. Consequently, it holds that ${F}_{{\rm{LC}}}=V\,f$. Here *V* stands for the volume of the LC body. The free energy density is expressed as

3 $$f={a}_{n}(T-{T}_{n}^{\ast }){S}^{2}-{b}_{n}{S}^{3}+{c}_{n}{S}^{4}+{a}_{s}(T-{T}_{s}^{\ast }){\eta }^{2}+{b}_{s}{\eta }^{4}+{c}_{s}{\eta }^{6}-DS{\eta }^{2}-{\varepsilon }_{0}{\rm{\Delta }}\varepsilon S{E}^{2}.$$

The quantities ${a}_{n},{b}_{n},{c}_{n},{T}_{n}^{\ast },{a}_{s},{b}_{s},{c}_{s},{T}_{s}^{\ast }$ are temperature independent material constants. The positive constant *D* determines the coupling strength between the nematic and smectic order parameter. The value of *D* monotonously increases with the length of aliphatic chains of nCB molecules. The sequence of LC phases and character of temperature driven phase transitions depends on the value of *D*. Qualitative changes in phase behaviour are obtained for the values $D={D}_{c}^{(1)} > 0$ and $D={D}_{c}^{(2)} > {D}_{c}^{(1)}$. On lowering *T* from the isotropic phase, the following behaviour is observed^26,29^. I-N-SmA phase sequence forms for $D\le {D}_{c}^{(2)}$ and the I-N phase transition is weakly 1^st^ order. On the other hand, the N-SmA phase transition is continuous for $D < {D}_{c}^{(1)}$ and discontinuous for ${D}_{c}^{(1)} < D < {D}_{c}^{(2)}$. For $D\ge {D}_{c}^{(2)}$, one observes a direct I-SmA discontinuous phase transition. Therefore, ${D}_{c}^{(1)}$ and ${D}_{c}^{(2)}$ correspond to the tricritical and to the I-N-SmA triple point, respectively. In the study, we consider 8CB and 12CB LCs, which are characterized by $D\sim {D}_{c}^{(1)}$ and $D\sim {D}_{c}^{(2)}$, respectively. The quantity ε_0_ is the electric permittivity constant and ${\rm{\Delta }}\varepsilon ={\varepsilon }_{\parallel }-{\varepsilon }_{\perp }$ measures the LC dielectric anisotropy, which is positive for nCB LCs. Here ${{\rm{\varepsilon }}}_{\parallel }$ and ${{\rm{\varepsilon }}}_{\perp }$ determine the dielectric response for the external field $\mathop{E}\limits^{\rightharpoonup }$ aligned parallel and perpendicular to $\mathop{n}\limits^{\rightharpoonup }$, respectively.

For result presentation and scaling purposes, we introduce the nematic uniaxial correlation length *ξ*_*n*_ and the nematic external field extrapolation length *ξ*_*e*_. They are temperature dependent and we express them at the nematic-isotropic phase transition temperature *T* = *T*_IN_ in the absence of an external electric field as

4 $${\xi }_{n}=\sqrt{\frac{{L}_{0}}{{a}_{n}({T}_{{\rm{IN}}}-{T}_{n}^{\ast })}},\,{\xi }_{e}=\sqrt{\frac{{L}_{0}\,{S}_{0}}{{\varepsilon }_{0}{\rm{\Delta }}\varepsilon {E}^{2}}},$$

here ${S}_{0}=S({T}_{IN})$ stands for the equilibrium value of the uniaxial order parameter at the I-N phase transition, and *L*_0_ determines the bare (temperature independent) nematic elastic constant in the single nematic elastic constant approximation. This constant determines elastic resistance to spatial non-homogeneities in nematic ordering. The quantity *ξ*_*n*_ estimates typical length on which the locally perturbed nematic order parameter recovers its bulk value at $T={T}_{{\rm{IN}}}$. On the other hand, *ξ*_*e*_ describes the typical distance on which locally perturbed nematic director field $\mathop{n}\limits^{\rightharpoonup }$ recovers orientation along $\mathop{E}\limits^{\rightharpoonup }$. In typical LCs it holds ${\xi }_{n}\sim 20$ nm, and ${\xi }_{e}\sim 0.1$ μm for $E\sim 10\,{\rm{kV}}\,{{\rm{cm}}}^{-1}$.

In terms of these experimentally measurable quantities, we express the following scaled and dimensionless quantities^1^:

5 $$\tilde{s}=\frac{S}{{S}_{0}},\tilde{\eta }=\frac{\eta }{{\eta }_{0}},r=\frac{T-{T}_{n}^{\ast }}{{\rm{\Delta }}{T}_{0}},{r}_{s}=\frac{T-{T}_{s}^{\ast }}{{T}_{s}^{\ast }},\tilde{D}=\frac{D{\eta }_{0}^{2}}{{a}_{n}{S}_{0}{\rm{\Delta }}{T}_{0}},\sigma ={(\frac{{\xi }_{n}}{{\xi }_{e}})}^{2},$$

where

$${\rm{\Delta }}{T}_{0}={T}_{{\rm{IN}}}-{T}_{n}^{\ast }\,{\rm{and}}\,{\eta }_{0}=\sqrt{{a}_{s}{T}_{s}^{\ast }/{b}_{s}}.$$

In this scaling, the dimensionless free energy density $\tilde{f}=f/({a}_{0}{S}_{0}^{2}{\rm{\Delta }}{T}_{0})$ reads

6 $$\tilde{f}\sim r{\tilde{S}}^{2}-2{\tilde{S}}^{3}+{\tilde{S}}^{4}-\sigma \tilde{S}+A({r}_{s}{\tilde{\eta }}^{2}+{\tilde{\eta }}^{4}/2)-\tilde{D}\tilde{S}{\tilde{\eta }}^{2},$$

where

$$A=\tfrac{{a}_{n}({T}_{IN}-{T}_{n}^{\ast }){S}_{0}^{2}}{{a}_{s}{T}_{s}^{\ast }{\eta }_{0}^{2}}.$$

We first summarise the main phase behaviour features in the absence of smectic ordering (more details are presented in the Supplementary). For *σ* = 0, the phase transition temperature *T*_IN_, the isotropic supercooling temperatures ${T}_{n}^{\ast }$, and the nematic superheating temperature ${T}_{n}^{\ast \ast }$, correspond to ${r}_{{\rm{IN}}}=r[{T}_{{\rm{IN}}}]=1$, ${r}^{\ast }=r[{T}_{n}^{\ast }]=0$, and ${r}^{\ast \ast }=r[{T}_{n}^{\ast \ast }]=9/8$, respectively. On increasing *σ*, the phase transition temperature increases as ${r}_{{\rm{IN}}}[\sigma ]=1+\sigma$ in the subcritical regime $\sigma  < {\sigma }_{c}=0.5$. In the supercritical regime $\sigma \ge {\sigma }_{c}$, the phase transition ceases to exist.

**Figure 1:**
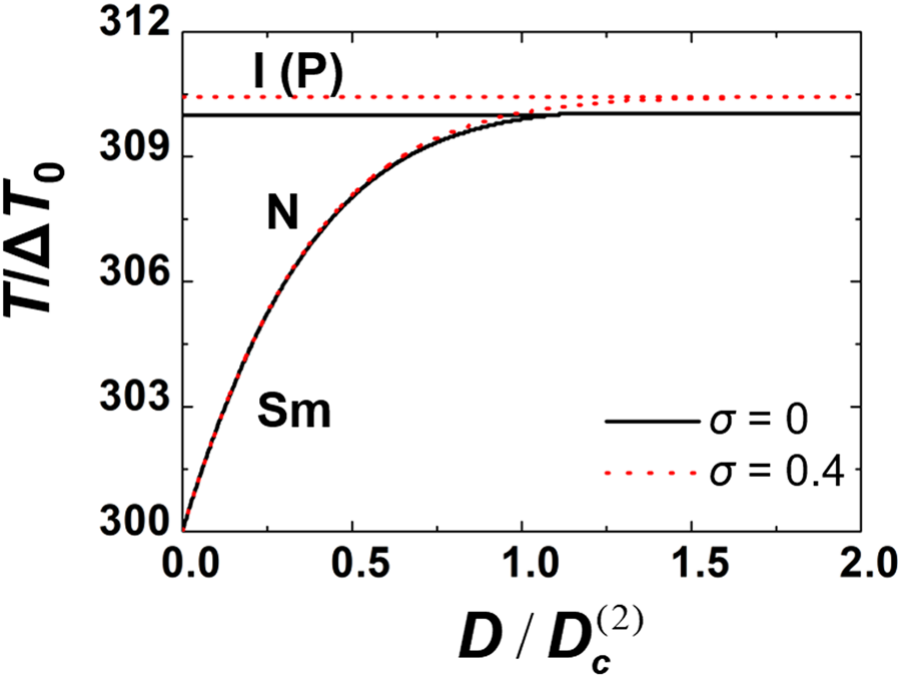
Stability regime of LC phases on varying the coupling strength *D* between the nematic and smectic order parameter for different values of the external electric field *E*, which is represented by the dimensionless parameter $\sigma \propto {E}^{2}$. The quantity ${D}_{c}^{(2)}$ corresponds to the triple point value of *D* calculated for *σ* = 0. For *σ* = 0.4 the phase transition temperatures are shifted to larger values and the isotropic phase is replaced by the paranematic (P) phase. In the calculations we set ${T}_{n}^{\ast }=309\,{\rm{K}}$, ${T}_{IN}=310\,{\rm{K}}$, ${T}_{s}^{\ast }=300\,{\rm{K}}$, *A* = 1.

Typical nematic and smectic phase behaviour resulting from equation (6) on varying *D* is depicted in Fig. 1. On decreasing temperature, we observed the phase sequence I-N-SmA for $D < {D}_{c}^{(2)}$ and I-SmA for $D\ge {D}_{c}^{(2)}$. If the external electric field is present the phase transition temperatures are increased. Furthermore, the isotropic phase is replaced by paranematic (P) ordering, which exhibits a finite degree of orientational ordering. Note that discontinuous I-N and P-N phase transitions are replaced by gradual evolution of orientational ordering on varying *T* for $\sigma  > 0.5$.

### ECE response: numerical analysis

We determined conditions maximizing Δ*T*_EC_ in nCB LCs. For this purpose, we first analyse the EC response in nematic ordering. The equations determining Δ*T*_EC_ are described in Methods (see equation (19)). In deriving them, we assume that i) the total entropy of the system is zero on adiabatically switching on or off *E*, and ii) that at each temperature, nematic ordering is determined by the Euler-Lagrange equilibrium equation. Therefore, we assume that the nematic order relaxation time is much shorter in comparison to the characteristic thermal relaxation time. The resulting expressions (see equation (17)) suggest that large responses are expected if one switches on *E* just above *T*_IN_, and *E* should be strong enough to induce nematic ordering. In such a way, the external field *E* driven increase in nematic ordering is large and consequently, so is the change in orientationally dependent entropy contribution.

**Figure 2:**
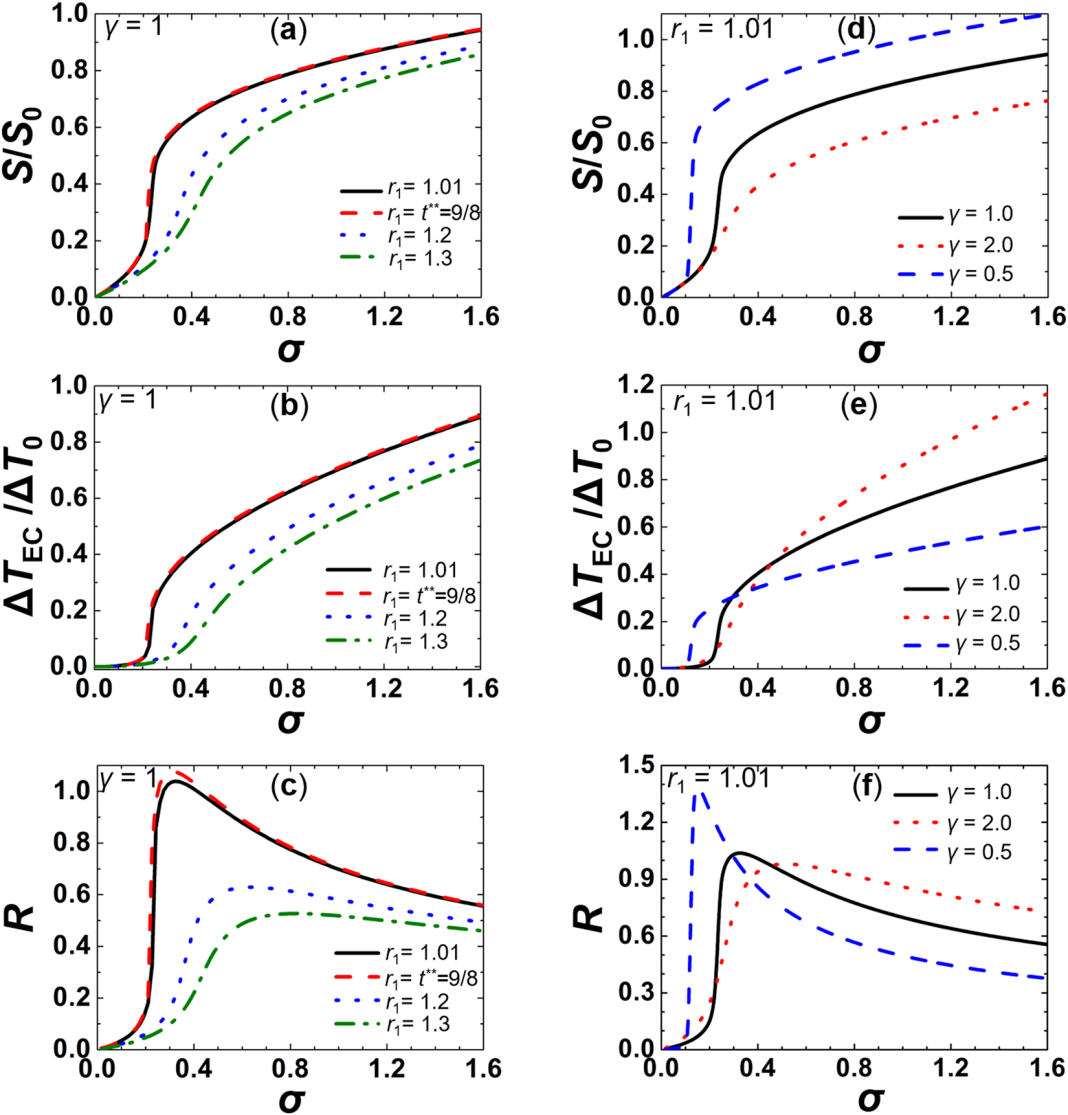
External field driven ECE responses on varying *T*_1_ (i.e. the scaled temperature ${r}_{1}=({T}_{1}-T)/{\rm{\Delta }}{T}_{0}$) (**a**–**c**) or γ (**d**–**f**). (**a**) *S* = *S*[σ], (**b**) ${\rm{\Delta }}{T}_{{\rm{EC}}}={\rm{\Delta }}{T}_{{\rm{EC}}}[\sigma ]$, (**c**) $R=R$[σ] for *γ* = 1. *r*_1_ = 1.01: black full curve; ${r}_{1}={r}^{\ast \ast }=9/8$ red dashed curve; ${r}_{1}=1.2$: blue dotted curve; ${r}_{1}=1.3$: green dash - dotted curve. (**d**) $S=S$[σ], (**e**) ${\rm{\Delta }}{T}_{{\rm{EC}}}={\rm{\Delta }}{T}_{{\rm{EC}}}[\sigma ]$, (**f**) *R* = *R*[σ] for ${r}_{1}=1.01$, $\gamma =0.5$: black full curve; $\gamma =1$: red dotted curve; $\gamma =2$: blue dashed curve ${S}_{0}=S[{T}_{IN},\sigma =0]$.

For this purpose, we considered temperatures just above the I-N phase transition. Representative results following the adiabatic switching on the external electric field are shown in Fig. 2. In this case $\{{E}_{1}=0,{T}_{1} > {T}_{IN},{S}_{1}=0\}$ and $\{{E}_{2}=E,{T}_{2}+{\rm{\Delta }}{T}_{{\rm{EC}}} > {T}_{1},S\equiv {S}_{2} > 0\}$. We plot $S=S[{\rm{\sigma }}]$, ${\rm{\Delta }}{T}_{{\rm{EC}}}={\rm{\Delta }}{T}_{{\rm{EC}}}[\sigma ]$ and $R=R[{\rm{\sigma }}]$ dependencies, where $R={\rm{\Delta }}{T}_{{\rm{EC}}}/\sigma {\rm{\Delta }}{T}_{0}$ is defined as the ratio of the temperature change with respect to the applied external electric field strength. We analyse the external field driven ECE responses on varying the initial temperature $T={T}_{1} > {T}_{{\rm{IN}}}[E=0]$ and material properties, which are collected in the dimensionless parameter γ (see equation (20)). In Fig. 2(a–c) we vary *T*_1_ (i.e. ${r}_{1}=({T}_{1}-{T}_{n}^{\ast })/({T}_{{\rm{IN}}}-{T}_{n}^{\ast })$ for fixed material properties, represented by *γ* = 1. Such values of *γ* roughly correspond to 8CB LC. One sees that on decreasing *T*_1_ towards *T*_*I*N_, *S*[σ] and ${\rm{\Delta }}{T}_{{\rm{EC}}}[\sigma ]$ display increasingly steeper s-shaped profiles. The efficiency of the response on applying the external field is evident from Fig. 2c, where the EC responsivity *R*[σ] is depicted. For low enough values of *T*_1_, the *R*[σ] dependence exhibits a well-pronounced maximum at *σ* < 0.4. For $\gamma =1$, these responses are subcritical.

Next, we analyse the behaviour of the above quantities on varying *γ*. We consider a temperature *T*_1_ close to *T*_IN_ where the EC responses are relatively large, and we analyse how the EC response could be further increased by choosing appropriate material properties. The resulting external field driven responses on varying γ are plotted in Fig. 2(d–f). One sees that on decreasing *γ*, the *S*[*σ*]and ${\rm{\Delta }}{T}_{{\rm{EC}}}[\sigma ]$ dependencies become steeper and below some critical value exhibit a 1^st^ order-type phase transition. It is evident that despite a relatively weaker nematic contribution in entropy, the EC response Δ*T*_EC_ could be larger, due to the observed discontinuous response. Figure 2f shows the EC responsivity *R* as a function of an external field σ. For *γ* = 0.5 it exhibits a sharp anomaly at *σ* ~ 0.2.

**Figure 3:**
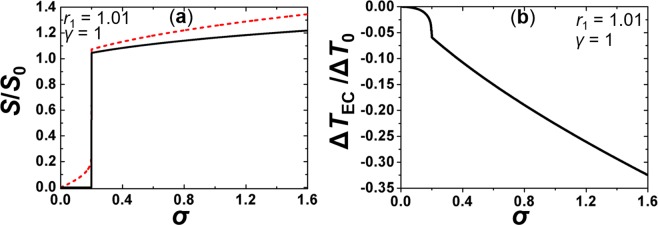
External field driven ECE responses when the field is switched off. (**a**) ${S}_{1}={S}_{1}$[σ] (red dashed curve) and ${S}_{2}={S}_{2}$[σ] (black full curve), (**b**) ${\rm{\Delta }}{T}_{{\rm{EC}}}={\rm{\Delta }}{T}_{{\rm{EC}}}[\sigma ]$. $\gamma =1$, ${r}_{1}=1.01$.

Finally, we consider the EC response after the external field is adiabatically switched off. For this purpose, we assume *E* is switched on for a long enough time so that the initial temperature *T* ~ *T*_1_ recovers. Then, we switch the field off and show the corresponding response in Fig. 3. In this case $\{{E}_{1}=E,{T}_{1} > {T}_{{\rm{I}}{\rm{N}}},{S}_{1} > 0\}$ and $\{{E}_{2}=0,{T}_{2}+{\rm{\Delta }}{T}_{{\rm{EC}}} < {T}_{1},{S}_{2} > 0\}$. The EC response is, in this case, weaker because both *S*_1_ and *S*_2_ are positive.

This analysis reveals that to achieve large EC responses on switching on *E*, the initial temperature should be just above the phase transition temperature. Note that the latent heat *L* of the phase transition for *E* = 0 is given by $L={{\rm{\Delta }}{\rm{\Omega }}}_{{\rm{LC}}}{T}_{IN}={a}_{n}{T}_{IN}V({S}_{+}^{2}-{S}_{-}^{2})={a}_{n}{T}_{IN}V{S}_{0}^{2}$, where ${S}_{-}=0$ (${S}_{+}={S}_{0}$) describes a value of *S* just below (above) *T* = *T*_*IN*_. By comparing the expression for *L* and equation (13), one sees that *L* dominates the Δ*T*_EC_ response for ${T}_{1}\sim {T}_{IN}$, providing $S[{T}_{2},E]\sim {S}_{0}$. Therefore, on increasing *L*, one expects a stronger EC response. The latter is expected to be larger at a direct I-SmA phase transition. The established smectic layers have a similar impact on nematic ordering as an ordering field. To estimate this effect, we compare the entropy changes in I-N and direct I-SmA phase transitions (details are given in Methods). Our derivation yields

7 $$\frac{{{\rm{\Delta }}{\rm{\Omega }}}_{{\rm{LC}}}[D={D}_{c}^{(2)}]}{{{\rm{\Delta }}{\rm{\Omega }}}_{{\rm{LC}}}[D < {D}_{c}^{(2)}]}=1+\tilde{A}\frac{{T}_{{\rm{IN}}}-{T}_{n}^{\ast }}{{T}_{{\rm{ISmA}}}-{T}_{s}^{\ast }},$$

where $\tilde{A}$ > 0 measures the ratio of representative smectic to nematic condensation penalties (see equation (13)). Here (i) ${{\rm{\Delta }}{\rm{\Omega }}}_{{\rm{LC}}}[D={D}_{c}^{(2)}]$ and (ii) ${{\rm{\Delta }}{\rm{\Omega }}}_{{\rm{LC}}}[D < {D}_{c}^{(2)}]$ describe the change in the LC contribution to the entropy for (i) the direct I-SmA phase transition at the triple point and (ii) I-N phase transition. Equation (7) suggests that in the former case, the latent heat is larger and its relative strength with respect to I-N change increases linearly with $\tilde{A}$.

### ECE response: experimental results

**Figure 4:**
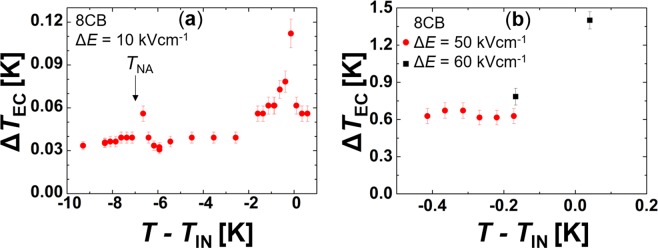
ECE response in 8CB LCs. On lowering the temperature in 8CB, the system exhibits I-N and N-SmA phase transitions at temperatures ${T}_{{\rm{IN}}}\sim 313.342\,{\rm{K}}$ and ${T}_{{\rm{NA}}}\sim 307\,{\rm{K}}$, respectively. (**a**) ${\rm{\Delta }}E=10\,{\rm{kV}}\,{{\rm{cm}}}^{-1}$, (**b**) ${\rm{\Delta }}E=50\,{\rm{kV}}\,{{\rm{cm}}}^{-1}$ and ${\rm{\Delta }}E=60\,{\rm{kV}}\,{{\rm{cm}}}^{-1}$.

**Figure 5:**
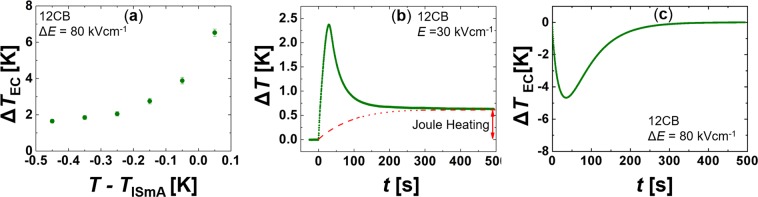
(**a**) The EC response in 12CB which exhibits the direct I-SmA phase transitions at ${T}_{{\rm{ISmA}}}\sim 332\,{\rm{K}}$. ${\rm{\Delta }}E=80\,{\rm{kV}}\,{{\rm{cm}}}^{-1}$. (**b**) A change in sample temperature Δ*T* as a function of time immediately after the external electric field is switched on at *t* = 0. The contribution due to the Joule heating is depicted with the dashed line. (**c**) The change in sample temperature Δ*T* as a function of time immediately after the external electric field is switched off at *t* = 0.

Our theoretical and numerical analysis indicates that large ECE responses are expected near the I-N phase transition. Furthermore, the derived equations suggest that the latent heat *L* released on entering the isotropic phase strongly influences Δ*T*_EC_. Therefore, LCs exhibiting a large value of *L* are expected to be advantageous as EC working materials. To verify these predictions, we measured Δ*T*_EC_ in (i) 8CB and (ii) 12CB LCs of the nCB family. They exhibit latent heats (i) $L\sim 5$ J/g at the N-I phase transition in 8CB and (ii) $L\sim 10$ J/g at the SmA-I phase transition in 12CB. We first measured the ECE response in 8CB using a moderate external field change Δ*E* = 10 kV cm^−1^ in a broad temperature range encompassing the SmA-N and I-N phase transition to probe where the responses are largest. Results shown in Fig. 4a reveal elevated Δ*T*_EC_ responses at the SmA-N (${\rm{\Delta }}{T}_{{\rm{EC}}}/{\rm{\Delta }}E\sim 6\times {10}^{-3}\,{\rm{K}}\,\mathrm{cm}/\mathrm{kV}$) and N-I $({\rm{\Delta }}{T}_{{\rm{EC}}}/{\rm{\Delta }}E\sim 8\times {10}^{-3}\,{\rm{K}}\,\mathrm{cm}/\mathrm{kV})$ phase transition. In line with the theoretical prediction, the response is larger in the latter transition due to the larger *E*-driven change in orientational ordering. Afterwards, we probed ECE responses at several temperatures using larger Δ*E* changes, see Fig. 4b. We obtained the maximal response ${\rm{\Delta }}{T}_{EC}\sim 1.4\,{\rm{K}}$ at $T\sim {T}_{IN}$ for Δ*E* = 10 kV cm^−1^, i.e. ${\rm{\Delta }}{T}_{{\rm{EC}}}/{\rm{\Delta }}E\sim 2\times {10}^{-2}\,{\rm{K}}\,\mathrm{cm}/\mathrm{kV}$. Finally, we measured Δ*T*_EC_ in 12CB, which exhibits a larger *L* value at the orientational order-disorder phase transition compared to 8CB. Figure 5a reveals that on approaching the phase transition, the ECE response monotonously increases. We obtained the largest response ${\rm{\Delta }}{T}_{{\rm{E}}{\rm{C}}}\sim 6.5\,{\rm{K}}$ (i.e. ${\rm{\Delta }}{T}_{{\rm{EC}}}/{\rm{\Delta }}E\sim 8\times {10}^{-2}\,{\rm{K}}\,\mathrm{cm}/\mathrm{kV}$) just above the SmA-I phase transition. Note that so far, this is the largest measured value Δ*T*_EC_ in LCs in general. In Fig. 5b we show a typical time evolution of the temperature in a liquid crystal when an external field is stepwise switched on. Initially, there is a relatively abrupt rise due to ECE. It is followed by roughly exponential decay towards a temperature plateau which is above the initial sample temperature. This temperature increase arises due to the Joule heating produced by ionic impurities in the sample. Figure 5c presents a typical response when an external field is switched off. In this case, we have a relatively abrupt temperature decrease due to ECE, followed by roughly exponential decay towards a temperature plateau.

## Conclusions

We study electrocaloric (EC) response in the nCB LC family both theoretically and experimentally. We focus on discontinuous order-disorder Isotropic-Nematic (I-N) or Isotropic-SmA (I-SmA)) phase transitions which display relatively large changes in orientational order entropy contribution. In our modelling, we used a mesoscopic Landau-de Gennes-Ginzburg approach in terms of nematic and smectic order parameters. The equations determining Δ*T*_EC_ reveal that latent heat dominates the EC response if an external field is adiabatically applied just above the phase transitions. Furthermore, calculations reveal that Δ*T*_EC_ monotonously increases if an initial sample temperature is approached from above the phase transition temperature. Furthermore, we analysed the impact of material properties and found that for $\gamma \le 1$, samples could exhibit discontinuous *E*-driven changes that are otherwise gradual. We also demonstrate analytically that the phase transition latent heat is significantly increased if the N-I phase transition is replaced by a direct SmA-I phase transition. For the nCB series, this takes place above the N-SmA-I triple point, i.e., for higher nCB homologues with n ≥ 10. We tested our theoretical and numerical predictions experimentally by measuring Δ*T*_EC_ using high precision calorimetry in 8CB and 12CB. These nCB representatives exhibit SmA-N-I and direct SmA-I phase transitions at increasing temperatures. Furthermore, the nematic-smectic coupling constant of 12CB is close to the tricritical point, which we used in our derivation where we express the latent heat at the direct SmA-I phase transition. Using relatively small changes in an external electric field, Δ*E* = 10 kV cm^−1^, we probed Δ*T*_EC_ response in 8CB over a wide temperature interval including both SmA-N and N-I phase transitions. Experiments reveal anomalous Δ*T*_EC_ increase at the transitions. In line with expectations, the response at N-I is larger due to the larger change in orientational ordering. For larger electric-field changes (Δ*E* = 50 kV cm^−1^ and Δ*E* = 60 kV cm^−1^) that are sufficient to induce complete crossing the I-N transition line and to fully release the latent heat, an enhanced ${\rm{\Delta }}{T}_{{\rm{EC}}}\sim 1.4\,{\rm{K}}$ is observed. This value, which is about 0.8 K above the electrocaloric response further away from the transition, is in good agreement with expected enhancement due to the released latent heat. In general, the measured responses are in line with our calculations, which suggest ${\rm{\Delta }}{T}_{{\rm{EC}}}\sim 1.4\,{\rm{K}}$ at order ${\rm{\Delta }}E\sim {\rm{V}}$ μm^−1^ just above I-N phase transition. In order to demonstrate the impact of the latent heat *L* on Δ*T*_EC_, we study ECE response in 12CB, which has roughly six times larger *L* than 8CB. At the SmA-I phase transition, we obtained ${\rm{\Delta }}{T}_{{\rm{EC}}}\sim 6.4\,K$ at Δ*E* = 80 kV cm^−1^ (${\rm{\Delta }}{T}_{{\rm{EC}}}/{\rm{\Delta }}E\sim 8\times {10}^{-8}\,{\rm{K}}\,\mathrm{cm}/\mathrm{kV}$), corresponding to the largest Δ*T*_EC_ measured so far in liquid crystals. The measured responses (both in 8CB and 12CB) are large enough to be exploited in potential EC-based applications. The temperature span in cooling devices could be further enhanced by an order of magnitude by regeneration techniques^16^. As an example, we present in the Supplementary an EC active regeneration mechanism. By commuting LC EC material between regions with and without an external electric field, one could dynamically build up a temperature difference between these parts of the device. In such devices, the liquid properties of LC electrocaloric coolant might be advantageous as LC material can play a role as an active regenerator. In contrast to current regeneration-based EC devices that exploit EC-passive regeneration material, devices exploiting an LC EC-active regenerator could significantly improve cooling power/mass ratio. Note that the obtained EC responses in LCs are comparable to those measured in competitive solid dielectric materials^30–32^. However, introducing LCs as EC material might open the gates to several new EC-based applications. In addition to liquid LC behaviour, we stress a rich vocabulary of different LC phases and structures. For example, using appropriate LC mixtures, one can tune temperature phase behaviour to desired regimes. Furthermore, EC-desired properties could be further tuned by doping LCs with appropriate nanoparticles (to reduce Joule heating or increase the phase transition temperature span).

## Methods

### Phenomenological model

In our theoretical and numerical analysis, we use a Landau-de Gennes-Ginzburg –type phenomenological mesoscopic approach. We describe the uniaxial orientational ordering by the nematic tensor order parameter^1^

8 $${\bf{Q}}=S(\mathop{n}\limits^{\rightharpoonup }\otimes \mathop{n}\limits^{\rightharpoonup }-\frac{1}{3}{\bf{I}}).$$

Here ⊗ stands for the tensor product and **I** is the identity tensor. The smectic A translational ordering is described by the smectic complex order parameter field

9 $$\psi =\eta {e}^{i\varphi }.$$

The amplitudes *S* and *η* determine the degree of orientational and translational ordering, respectively; the symmetry breaking fields $\mathop{n}\limits^{\rightharpoonup }$ and *ϕ* determine the structure of nematic and smectic ordering.

In terms of order parameter fields, we express the free energy density as a sum $f={f}_{c}^{(n)}+{f}_{c}^{(s)}+{f}_{e}+{f}_{f}+{f}_{coupl}$, consisting of the nematic condensation (${f}_{c}^{(n)}$), smectic condensation (${f}_{c}^{(n)}$), elastic (*f*_*e*_), external electric field (*f*_*f*_), and coupling (*f*_*coupl*_) contributions. These terms are expressed as

10a $${f}_{c}^{(n)}=\frac{3{a}_{n}}{2}(T-{T}_{n}^{\ast })Tr{{\bf{Q}}}^{2}-\frac{9{b}_{n}}{2}Tr{{\bf{Q}}}^{3}+\frac{9{c}_{n}}{4}{(Tr{{\bf{Q}}}^{2})}^{2},$$

10b $${f}_{c}^{(s)}={a}_{s}(T-{T}_{s}^{\ast })\,|\psi {|}^{2}+{b}_{s}|\psi {|}^{4}+{c}_{s}|\psi {|}^{6},$$

10c $${f}_{e}={L}_{0}|\nabla {\bf{Q}}{|}^{2}+{C}_{\perp }|\mathop{n}\limits^{\rightharpoonup }\times \nabla \psi {|}^{2}+{C}_{\parallel }|(\nabla -i{q}_{0}\mathop{n}\limits^{\rightharpoonup })\psi {|}^{2},$$

10d $${f}_{f}=-\,\frac{3{\varepsilon }_{0}{\rm{\Delta }}\varepsilon }{2}\mathop{E}\limits^{\rightharpoonup }\cdot {\bf{Q}}\mathop{E}\limits^{\rightharpoonup },$$

10e $${f}_{coupl}=-\,\frac{3D}{2}\nabla \psi \cdot {\bf{Q}}\nabla {\psi }^{\ast }.$$

The condensation terms ${f}_{c}^{(n)}$ and ${f}_{c}^{(s)}$ determine the equilibrium value of the nematic and smectic order parameter if the order parameters are decoupled, i.e. ${f}_{coupl}=0$. For positive material constants *a*_*n*_, *b*_*n*_, *c*_*n*_, *a*_*s*_, *b*_*s*_, *c*_*s*_ and *D* = 0, the bulk of I-N and N-SmA phase transitions are of the first and second order, realized at ${T}_{{\rm{IN}}}={T}_{n}^{\ast }+\frac{{b}_{n}^{2}}{4{a}_{n}{c}_{n}}$ and $T={T}_{s}^{\ast }$, respectively. The elastic contribution *f*_*e*_ is weighted by the positive representative bare nematic (*L*_0_), smectic bend (${C}_{\perp }$) and smectic compressibility (*C*_||_) elastic constant. We henceforth set ${C}_{0}\equiv {C}_{\parallel }\sim {C}_{\perp }$ and neglect the smectic elastic anisotropy. This term penalizes spatial inhomogeneities in order parameters *S* and *η*, favours homogeneous ordering of $\mathop{n}\limits^{\rightharpoonup }$ along a single symmetry breaking direction, tends to align the smectic layer normal $\mathop{{\nu }_{s}}\limits^{\rightharpoonup }=\nabla \varphi /|\nabla \varphi |$ along $\mathop{n}\limits^{\rightharpoonup }$ and enforces the layer distance $d=2\pi /{q}_{0}$. The field term *f*_*f*_ describes the coupling of nematic order parameter with the external electric field *E*. We consider LCs with positive dielectric anisotropy Δ*ε*, and *ε*_0_ stands for the dielectric permittivity constant. The N-SmA order parameter coupling term *f*_*coupl*_ is weighted by the positive coupling constant *D*.

In our study we limit to the electrocaloric effect (ECE) in bulk where we neglect spatial inhomogeneities in order parameters. We set the parameters so that $\mathop{n}\limits^{\rightharpoonup }$ is homogeneously aligned along a single symmetry (say, along the *z-*axis) direction, the SmA layers adopt equilibrium spacing $d=2\pi /{q}_{0}$ and $\varphi ={q}_{0}z$. Furthermore, we set that $\mathop{E}\limits^{\rightharpoonup }$ is imposed along the *z*-axis. With this in mind, we obtained equation (3).

### The impact of smectic order on latent heat

Our aim is to demonstrate that the change in the orientationally dependent part of the entropy at the I-SmA phase transition is larger in comparison to the change at the I-N phase transition. For this purpose, we focus on a regime where the coupling between the nematic and smectic ordering is relatively strong, therefore $D\ge {D}_{c}^{(1)}$. We express the nematic ordering in the presence of smectic layers as the sum $S=s+\delta S$. Here *s* minimizes free energy density *f* for $\eta =0$, and *δS* measures increase in orientational ordering if smectic layers are present. Expanding *f* up to the second order in *δS* and minimizing the resulting expression with respect to *δS* for *E* = 0 yield*s*

11 $$f={a}_{n}(T-{T}_{n}^{\ast }){s}^{2}-{b}_{n}{s}^{3}+{c}_{n}{s}^{4}+{a}_{s}(T-{T}_{s}^{(eff)}){\eta }^{2}-{b}_{s}^{(eff)}{\eta }^{4}+{c}_{s}{\eta }^{6},$$

where ${T}_{s}^{(eff)}={T}_{s}^{\ast }+Ds/{a}_{s}$, ${b}_{s}^{(eff)}={D}^{2}\chi -{b}_{s} > 0$, and ${\chi }^{-1}=\frac{{\partial }^{2}f}{\partial {S}^{2}}[S=s]$. Hence, we obtain decoupled equations for *s* and *η* where both order parameters exhibit 1^st^ order phase transition at varying temperatures. In addition to *r*, we also introduce dimensionless smectic scaled temperatures ${r}_{s}=(T-{T}_{s}^{(eff)})/({T}_{c}-{T}_{s}^{(eff)})$, and scaled order parameters $\tilde{s}=S/{S}_{0}$ and $\tilde{\eta }=\eta /{\eta }_{0}$. Here *T*_*c*_ denotes the phase transition temperature at which the smectic ordering appears for $\sigma =0$, ${S}_{0}=S\,[T={T}_{{\rm{IN}}},\sigma =0]$, and ${\eta }_{0}=\eta [T={T}_{c},\sigma =0]$. It follows that

12 $$\tilde{f}=r{\tilde{s}}^{2}-2{\tilde{s}}^{3}+{\tilde{s}}^{4}+\tilde{A}({r}_{s}{\tilde{\eta }}^{2}-2{\tilde{\eta }}^{4}+{\tilde{\eta }}^{6}),$$

where $\tilde{f}=f/({a}_{n}({T}_{{\rm{IN}}}-{T}_{n}^{\ast }){S}_{0}^{2})$ and the coefficient $\tilde{A}$ measures the relative strength of nematic and smectic free energy condensation contributions:

13 $$\tilde{A}=\frac{{a}_{n}({T}_{IN}-{T}_{n}^{\ast }){S}_{0}^{2}}{{a}_{s}({T}_{c}-{T}_{s}^{(eff)}){\eta }_{0}^{2}}.$$

In this scaling at $T={T}_{{\rm{IN}}}$ it holds that $r=1$, $\tilde{s}=1$, and at *T* = *T*_*c*_ it follows ${r}_{s}=1$ and $\tilde{\eta }=1$.

We use equation (1) to calculate the orientational entropy change on crossing into the isotropic phase. In the isotropic phase it holds ${{\rm{\Omega }}}_{{\rm{LC}}}=0$; just below the phase transition line one obtains

14a $$\frac{{{\rm{\Omega }}}_{{\rm{LC}}}[D < {D}_{c}^{(2)}]}{{a}_{n}V}=-\,1,$$

14b $$\frac{{{\rm{\Omega }}}_{{\rm{LC}}}[D={D}_{c}^{(2)}]}{{a}_{n}V}=-\,(1+A\frac{{T}_{{\rm{IN}}}-{T}_{n}^{\ast }}{{T}_{{\rm{ISmA}}}-{T}_{s}^{\ast }}),$$

where $${T}_{c}[D={D}_{c}^{(2)}]={T}_{{\rm{ISmA}}}$$.

### EC response above *T*_IN_

We have also derived equations determining the electrocaloric response on adiabatically switching on or off the external field *E* in the nematic or isotropic phase. The initial and final system state are determined by {*E*_1_, *T*_1_} and {*E*_2_, *T*_2_}, respectively. In the adiabatic process, the total change of entropy equals zero, therefore it holds that

15 $${{\rm{\Delta }}{\rm{\Omega }}}_{{\rm{LC}}}={{\rm{\Delta }}{\rm{\Omega }}}_{l},$$

${{\rm{\Delta }}{\rm{\Omega }}}_{{\rm{LC}}}={{\rm{\Omega }}}_{{\rm{LC}}}[{E}_{2},{T}_{2}]-{{\rm{\Omega }}}_{{\rm{LC}}}[{E}_{1},{T}_{1}]$ and ${{\rm{\Delta }}{\rm{\Omega }}}_{l}={{\rm{\Omega }}}_{l}[{T}_{2}]-{{\rm{\Omega }}}_{l}[{T}_{1}]$. The change in the lattice entropy is given by equation (2). The orientational LC ordering contribution can be expressed as the partial derivative of the free energy density at a constant value of an external electric field:

16 $${{\rm{\Omega }}}_{{\rm{LC}}}=-\,{[\frac{\partial {F}_{LC}}{\partial T}]}_{E}=-\,{a}_{n}{S}^{2}V$$

Here we set that LC ordering attains the equilibrium ordering. Hence, we assume that the nematic order parameter relaxation time is much shorter with respect to the thermal relaxation time. This is a sensible assumption, because typical order parameter relaxation times close to I-N phase transition are of the order of $\tau \sim {10}^{-7}\,{\rm{s}}$. On the other hand, the characteristic thermal relaxation time is typically of the order of 1 ms in confinements exhibiting typical linear lengths of µm size^2^.

By combining equations (2), (15) and (16) we obtain a self-consistent equation for *T*_2_:

17 $${T}_{2}={T}_{1}\,{\exp }(\frac{{a}_{n}}{{C}_{l}}({S}_{2}^{2}-{S}_{1}^{2})),$$

where ${S}_{2}\equiv S[{E}_{2},{T}_{2}]$ and ${S}_{1}\equiv S[{E}_{1},{T}_{1}]$. Values of *S*_1_ and *S*_2_ are determined by the equilibrium equation $\partial f/\partial S=0$ for the states determined by (*E*_1_, *T*_1_) and (*E*_2_, *T*_2_), respectively.

We now consider cases where ${T}_{1}\equiv T > {T}_{{\rm{IN}}}$ and ${T}_{2}\equiv T+{\rm{\Delta }}{T}_{{\rm{EC}}}$. For cases ${\rm{\Delta }}{T}_{{\rm{EC}}}/T\ll 1$, equation (17) simplifies to

18 $${\rm{\Delta }}{T}_{{\rm{EC}}}/T\sim ({S}_{2}^{2}-{S}_{1}^{2})\frac{{a}_{n}}{{C}_{l}}.$$

Using dimensionless quantities, we express equation (18) describing the ECE response and the equilibrium equations for the degree of nematic ordering. It follows

19a $${r}_{2}-{r}_{1}-\gamma ({\tilde{s}}_{2}^{2}-{\tilde{s}}_{1}^{2})=0,$$

19b $$2{r}_{i}{\tilde{s}}_{i}-6{\tilde{s}}_{i}^{2}+4{\tilde{s}}_{i}^{3}-{\sigma }_{i}=0,$$

where *i* = {1, 2}, $${r}_{i}\equiv r[{T}_{i}],\,{\sigma }_{i}\equiv {\sigma }_{i}[{E}_{i}],\,{\tilde{s}}_{i}\equiv \tilde{s}[{r}_{i},{\sigma }_{i}],$$ and

20 $$\gamma =\frac{{a}_{n}{T}_{n}^{\ast }{S}_{0}^{2}}{{C}_{l}{\rm{\Delta }}{T}_{0}}.$$

Here we took into account that $r+{T}_{n}^{\ast }/{\rm{\Delta }}{T}_{0}\sim {T}_{n}^{\ast }/{\rm{\Delta }}{T}_{0}$. For a reference LC (5CB) close to *T*_IN_, it holds ${a}_{n}\sim {10}^{5}\,{{\rm{JK}}}^{-1}\,{{\rm{m}}}^{-3}$, ${T}_{n}^{\ast }\sim 300\,{\rm{K}}$, ${\rm{\Delta }}{T}_{0}\sim 1\,{\rm{K}}$, ${S}_{0}\sim 0.3$, and ${C}_{l}\sim 4\cdot {10}^{6}\,{\rm{J}}\,{{\rm{m}}}^{-3}$. Therefore, typically *γ* ~ 1 and ${T}_{n}^{\ast }/{\rm{\Delta }}{T}_{0} > 100$. Note, that it holds $\frac{{a}_{n}{S}_{0}^{2}}{{C}_{l}}=\gamma \frac{{\rm{\Delta }}{T}_{0}}{{T}_{n}^{\ast }}\sim 0.01$, which justifies expansion used in deriving equation (18). The equations were solved using the Newton method^33^.

### Experimental set up

In order to determine the ECE in soft materials, a high-resolution calorimeter was utilized with small modifications. Its exceptional temperature stability allowed measurements of each sample’s EC temperature variations induced by the external electric field.

We used octyl cyanobiphenyl (8CB) and dodecyanobiphenyl (12CB) LCs. On decreasing temperature, the 8CB exhibits the 1^st^ order I-N phase transition at ${T}_{{\rm{IN}}}\sim 313\,{\rm{K}}$. On further decreasing temperature, a weakly 1^st^ order nematic – smectic A (N-SmA) phase transition takes place at ${T}_{{\rm{NA}}}\sim 307\,{\rm{K}}$. In 12CB we have a direct I-SmA phase transition at ${T}_{{\rm{ISmA}}}\sim 332\,{\rm{K}}$.

A liquid crystal compound of about 5 mg–10 mg was loaded into a high-purity glass cell composed of two 140 μm thick glass plates, coated by indium tin oxide (ITO) electrodes and separated by a 120 μm thick Mylar spacer. The temperature of the cell was measured by a small-bead thermistor attached to the glass plate. The ECE measurement protocol is described in detail in refs^8,16^. In the standard protocol that was used in our experiments, step-like electric pulses were applied, always starting from 0 V. The duration of the electric pulses was long enough to allow the sample to reach thermal equilibrium with the surrounding bath. This time was typically much longer than the external thermal time of approximately 100 s. The typical internal thermal time scale required for the whole system to reach internal thermal equilibrium was about 20 s. The relaxation of the temperature of the whole system, composed of the cell, thermistor, glue, and attaching wires, was monitored on a time scale much longer than the time scale required for internal equilibration.

In the data analysis, the long temperature relaxation tail of the internally thermally equilibrated sample was fitted to the exponential decay ansatz $T(t)={T}_{B}+{\rm{\Delta }}{T}_{C}\,{\exp }^{-t/\tau }$ in order to determine temperature change Δ*T*_*C*_. In most cases, some Joule heating ${T}_{J}(t)={T}_{B}+{\rm{\Delta }}{T}_{J}(1-{\exp }^{-t/\tau })$ was present and was subtracted from the electrocaloric data. In such cases, the remaining result *T*(*t*) − *T*_*J*_(*t*), was fitted to the exponential decay ansatz in order to determine the true change of the cell temperature Δ*T*_*C*_ due to the ECE. This was later additionally corrected by taking into account all masses constituting the cell that were carefully measured prior to the experiment and which by absorbing the heat, during the internal heat equilibration, influenced the measured ECE temperature response ${\rm{\Delta }}{T}_{{\rm{EC}}}={\rm{\Delta }}{T}_{C}\,{\sum }_{i}\,{C}_{p}^{i}/{C}_{p}^{{\rm{EC}}}$. Here, ${C}_{p}^{i}={m}^{i}{c}_{p}^{i}$ represents the heat capacity of each constituent, i.e., the heat capacities of the sample, glass plates, thermistor, attaching wires, etc. ${C}_{p}^{{\rm{EC}}}$ stands for the heat capacity of the EC active material, i.e., the part of the LC sample under the electrodes.

## Supplementary information

Supplementary materials
