# Supplementary material for: Giant electrocaloric response in smectic liquid crystals with direct smectic-isotropic transition
Source: Sci Rep. 2019 Feb 11;9:1721. doi: 10.1038/s41598-019-38604-9 (PMC6370888; doi:10.1038/s41598-019-38604-9)
Supplement: Supplementary file 1 — Supplementary materials [file 41598_2019_38604_MOESM1_ESM.docx]

**Giant electrocaloric response in smectic liquid crystals with direct smectic-isotropic transition**

Klemenčič Eva^1^, Trček Maja^2^, Kutnjak Zdravko^2,3, *^, and Kralj Samo^1,2, *^

*^1^ Faculty of Natural Sciences and Mathematics, Koroška cesta 160, 2000 Maribor, Slovenia.*

*^2^ Jozef Stefan Institute, Jamova 39, 1001 Ljubljana, Slovenia.*

*^3^ The Jozef Stefan International Postgraduate School, Jamova 39, 1001 Ljubljana, Slovenia.*

**Supplementary information**

We first analyse the impact of an external electric ordering field *E* on the nematic - isotropic (paranematic) – phase transition in the absence of smectic ordering. Next, we schematically present a possible realization of an ECE – based cooling device and an active regeneration device.

1. ***Nematic phase behaviour***

The temperature behaviour of the scaled order parameter $\tilde{s}=S/S_{0}$ on varying $\sigma$, which minimizes the dimensionless free energy density *f* (see equation (6)) in the absence of smectic ordering, is depicted in Fig. S1. We use the scaling introduced in equation (5). In the absence of an external electric field ($\sigma$ = 0), the I-N phase transition occurs at $r_{\mathrm{IN}}=1$, where ${\tilde{s}(r}_{\mathrm{IN}})=1$. The scaled supercooling temperature equals $r^{*}=0$ and the superheating temperature, $r^{**}=9/8$. The equilibrium degree of order equals $\tilde{s}(r>r_{\mathrm{IN}})=0$ and $\tilde{s}\left( r<r_{\mathrm{IN}} \right)=\left( 3+\sqrt{9-8r} \right)/4.$

For $\sigma>0$, isotropic ordering is replaced by paranematic (P) ordering. The latter refers to a weakly orientationally ordered phase. On increasing $\sigma$, the paranematic-nematic (P-N) phase transition temperature ($r_{\mathrm{IN}}[\sigma]$) increases until the critical value $\sigma_{c}=0.5$ is reached. For ${\sigma<\sigma}_{c}$, it holds $\text{r}_{\text{IN}}\left[ \text{σ} \right]\text{=1+σ}$, corresponding to

|  | $T_{\mathrm{IN}}\left[ \sigma\right]-T_{\mathrm{IN}}=\Delta T_{0} \sigma$. | (S1) |
| --- | --- | --- |

At the phase transition temperature, the equilibrium degree of ordering equals $\tilde{s}\left( r_{\mathrm{IN}}[\sigma] \right)=\left( 1+\sqrt{1-2\sigma} \right)/2$. In the regime ${\sigma>\sigma}_{c}$, the P-N phase transition becomes supercritical. Therefore, at varying temperatures, the orientational ordering exhibits gradual changes.


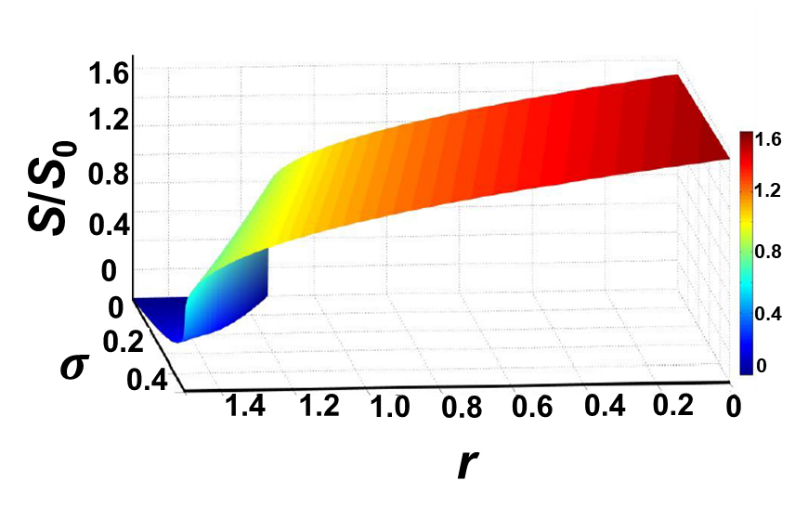


**Figure S1.** Temperature behaviour of $S/S_{0}$ on increasing $\sigma$ where $r=(T-T_{n}^{*})/\Delta T_{0}$. Colour reveals the degree of nematic ordering. Blue colour marks the isotropic ($S=0$) and the paranematic order ( $0<S\ll S_{0})$. The 1^st^ order P-N and phase transition temperature linearly increases with increasing field strength $\sigma$ < 0.5. For $\sigma$ ≥ 0.5 the supercritical regime is entered.

We next determine the bistability regime, where both N and P phases can exist for external fields ranging from $\sigma=0$ to $\sigma=0.5\equiv\sigma_{c}$. In Figure S2, we demonstrate superheating (red solid curve) and supercooling (blue dashed curve) responses in nematic ordering for two field strengths $\sigma=0.1$ and $\sigma=0.4$. The phase transition takes place at $r_{\mathrm{IN}}[\sigma]=1+\sigma$ (green thick curve). However, as the temperature gradually increases above $r_{\mathrm{IN}}[\sigma]$, nematic ordering could persist in a metastable state till $r^{**}\left[ \sigma\right].$ Alternatively, as the temperature gradually decreases, isotropic (paranematic) ordering could persist below $r_{\mathrm{IN}}[\sigma]$ till $r^{*}\left[ \sigma\right]$. The bistability regime in the $\left\{ r,\sigma\right\}$ plane is depicted in Figure S3.

**
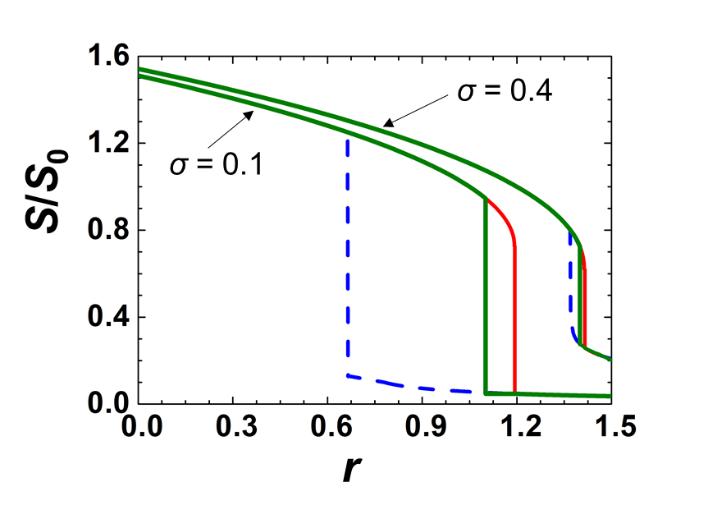
**

**Figure S2**. The scaled order parameter variation on increasing and decreasing temperature $r=(T-T^{*})/\Delta T_{0}$ for external field strengths $\sigma=0.1$ and $\sigma=0.4$. The corresponding phase transition temperatures are equal to $r_{\mathrm{IN}}[0.1]=1.1$ and $r_{\mathrm{IN}}[0.4]=1.4$, respectively. Equilibrium dependencies are plotted with green solid thick curves, supercooled states with blue dashed curves, and superheated states with red solid curves.

**
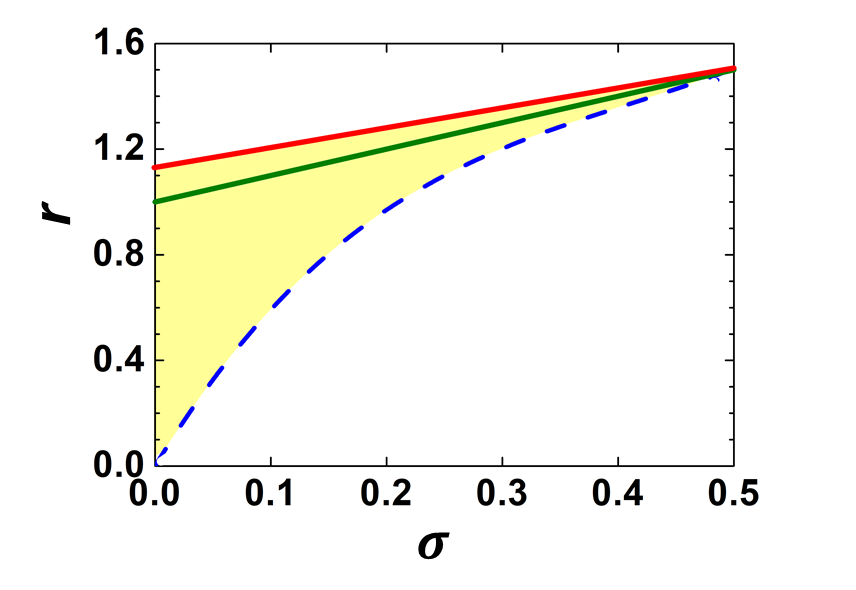
**

**Figure S3**. Supercooling ($r^{*}[\sigma]$: blue dashed curve), superheating ($r^{**}$[$\sigma$]: red solid curve) and phase transition ($r_{\mathrm{IN}}$[$\sigma$]: green solid thick curve) temperatures as functions of an external dimensionless field strength $\sigma$. The region of bistability (yellow coloured) shows where N and P phases can coexist.

1. ECE based applications

We propose two different applications based on ECE. The corresponding schematic sketches are depicted in Fig. S4 and Fig. S5.

We first consider an ECE based refrigerator. The basic working unit is depicted in Fig. S4a. The LC component is sandwiched between two thermal reservoirs, labelled A and B. They exhibit temperatures $T_{A}$ and $T_{B}>T_{A}$, respectively. Furthermore, between each thermal reservoir i={A,B} and LC unit we place thermal diodes, which we label in Fig. S4a as D_i_. We assume that they act as thermal isolators (conductors) if a strong enough voltage is applied to them.


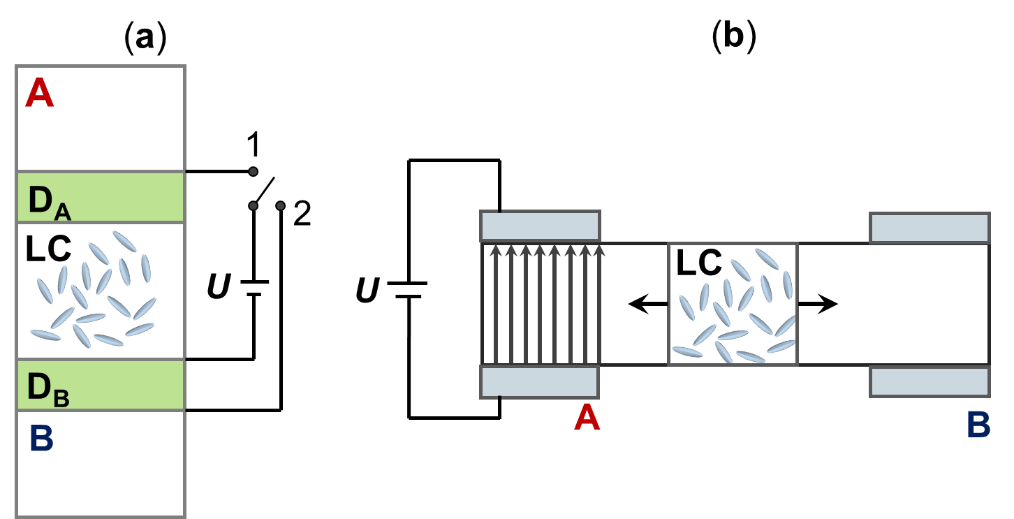


**Figure S4.** Schematic representations of ECE based devices. (**a**) The EC material sandwiched between temperature isolating/conducting layers (green shadowed elements in the figure). These layers act as an isolator (conductor) when an external field is switched on (off). They separate the EC material with thermal reservoirs A and B, exhibiting temperatures $T_{A}$ and $T_{B}$, respectively. The reservoir A (B) is thermally coupled with the EC material when the switcher is in position 2 (1). (**b**) The EC material alternates between the left (A) and right (B) regions, exhibiting  $T\sim T_{A}$, and $T\sim T_{B}$, respectively.

Figures S5a-S5d demonstrate a cycle in which heat is extracted from A and we assume $T_{A} >T_{\mathrm{IN}}$. Let us assume that LC initially exhibits $T\sim T_{A}$ (see Fig. S6 for times $0<t<t_{1}$). At time $t=t_{1}$ the switcher shown in Fig. S4a is closed on 1. Consequently, the external field is applied simultaneously to D_A_ and LC. We assume that the external field is adiabatically applied to LC, and D_B_ is thermally conducting. Hence, at $t\sim t_{1}$, the temperature of the LC unit is increased to $T=T_{A}+\Delta T_{\mathrm{EC}}>T_{B}$, (see Fig. S6 and Fig. S5a). With time, the heat flows to B and the temperature of the LC unit gradually approaches $T_{B}$ (see Fig. S5b and Fig. S6 within the time window$(t_{1},t_{2}$)). At $t=t_{2}$ the switcher is closed on 2 (see Fig. S4a). We propose that the LC system adiabatically enters a disordered state which results in a temperature drop to $T=T_{B}-\Delta T_{\mathrm{EC}}<T_{A}$ (see Fig. S5c and Fig.S6 at $t=t_{2}$). Furthermore, the thermal diode D_A_ enters the conductive state. Therefore, with time, the LC unit extracts heat from A and its temperature gradually approaches $T\sim T_{A}$ (see Fig. S5d and Fig. S6 for $t>t_{2}$).

Next, we present a scheme which could serve as an active regenerator. Using it, one could establish a temperature difference by commuting an ECE unit using appropriate dynamics between regions with and without an external field. In Fig. S4b, we present a scheme in which the ECE unit commutes between region A, in which a strong enough external field $E$ is present, and region B, where the field is absent. In Figs. S5e-S5h, we show key stages of the active regenerator. Let us suppose that the whole system is initially at a constant temperature $T=T_{0}>T_{\mathrm{IN}}$. Therefore, for $E=0$ the LC unit exhibits the isotropic phase. At time $t=t_{1}$, we adiabatically shift the LC unit into region A, where the field aligns the nematic director field along its direction (Fig. S5e). Consequently, the temperature of the LC unit is increased to $T=T_{0}+\Delta T_{\mathrm{EC}}$, and it acts as a heat source. The LC unit remains in region A for a long enough time interval Δ*t*, that heat begins to flow from it into the surrounding region A, heating it to temperature $T_{A} =T_{0}+\Delta T_{A}$ (Fig. S5f). Then, we adiabatically shift the ECE unit into region B, see (Fig. S5g). LC converts into the isotropic phase and, due to the ECE, its temperature drops to $T\cong T_{A}-\Delta T_{\mathrm{EC}}<T_{0}$. The LC unit in region B acts now as a heat sink and it begins to extract heat from the surrounding B region, whose temperature drops to $T=T_{0}-\Delta T_{B}$. Therefore, in the initially thermally isotropic system at $T=T_{0}$ we have established the temperature difference $\Delta T_{AB}\cong{\Delta T}_{A}-\Delta T_{B}$ between regions A and B. This difference could be increased by repeating the cycle and choosing optimal commuting dynamics.


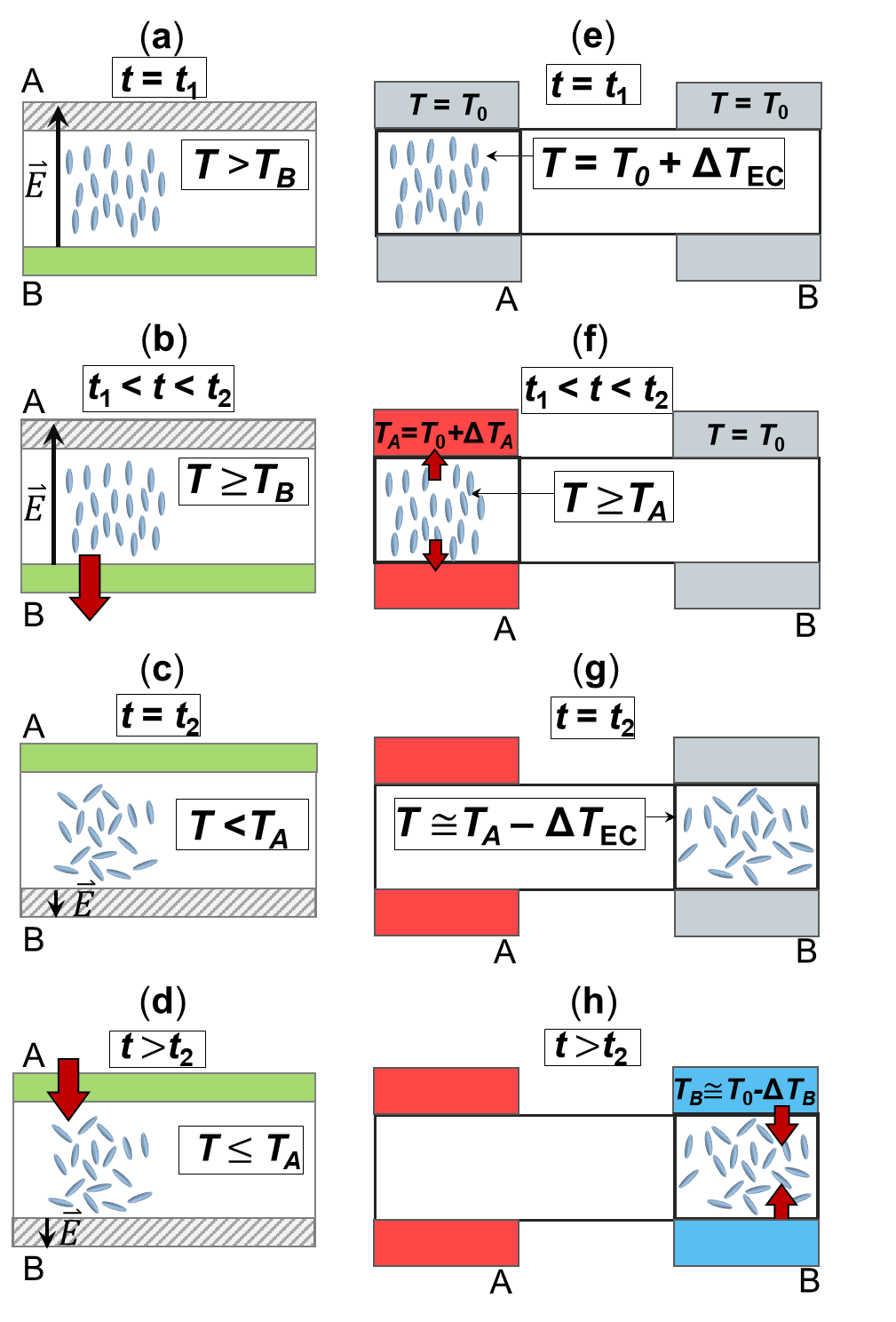


**Figure S5.** (**a-d**) The EC material sandwiched between thermal reservoirs A and B. Shadowed elements act as a heat isolator (conductor) if an external electric field is switched on (off) (see Figure S4a). (**e-h**) The EC material commuting between regions A and B. While in region A, the EC element experiences an external electric field (see Figure S4b).


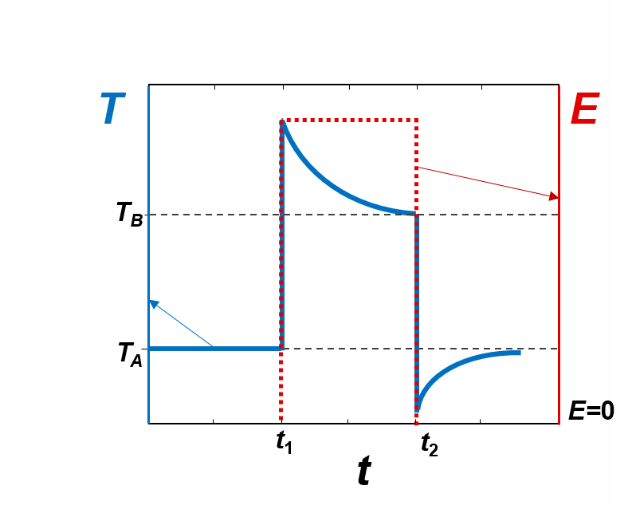


**Figure S6.** Temperature of the EC material as a function of time. At $t=t_{1}$, an external field is adiabatically applied to the EC material, and adiabatically switched off at $t=t_{2}$.
